# Mechanical comparison of cortical button fixation, interference screw and keyhole techniques in subpectoral biceps tenodesis, including digital image correlation assessment of bone surrounding the drill hole

**DOI:** 10.1002/jeo2.70313

**Published:** 2025-07-02

**Authors:** Lukáš Martinek, Petr Boháč, Vasileios Apostolopoulos, Tomáš Návrat, Róbert Langer, Luboš Nachtnebl, Tomáš Tomáš

**Affiliations:** ^1^ First Department of Orthopaedic Surgery, St. Anne's University Hospital and Faculty of Medicine Masaryk University Brno Czechia; ^2^ Institute of Solid Mechanics, Mechatronics and Biomechanics, Faculty of Mechanical Engineering University of Technology Brno Czechia

**Keywords:** biceps tenodesis, cortical button, digital image correlation, interference screw, keyhole technique, subpectoral tenodesis

## Abstract

**Purpose:**

Subpectoral biceps tenodesis is a widely used surgical technique to relieve pain and restore function in the shoulder by securing the long head of the biceps tendon. This study aimed to evaluate the mechanical performance of three fixation techniques using cortical button, interference screw and keyhole methods by assessing their strength, durability and strain distribution, incorporating the novel application of digital image correlation (DIC).

**Methods:**

Thirty fresh porcine bone‐tendon specimens were allocated evenly among the fixation techniques. Biomechanical testing involved cyclic axial loading (10–100 N) for 500 cycles, followed by load‐to‐failure testing using a universal testing machine. DIC analysis assessed strain distribution around the bone drill site. Statistical comparisons of displacement, load‐to‐failure and strain patterns were performed.

**Results:**

Cortical button fixation demonstrated the highest average load‐to‐failure at 353 ± 45 N, with all specimens completing 500 cycles and showing the least variability. In comparison, interference screw fixation had the lowest average load‐to‐failure (271 ± 71 N) with two failures occurring before 500 cycles, and the keyhole technique showed intermediate performance at 319 ± 45 N, also with two early failures. Cyclic displacement after 500 cycles was lowest for the interference screw (3.16 ± 0.52 mm), followed by the keyhole (11.51 ± 2.08 mm), and highest for the cortical button (13.84 ± 1.90 mm). Displacement range after 500 cycles was also lowest in the interference screw group (0.62 ± 0.05 mm), compared to the cortical button (0.88 ± 0.07 mm) and keyhole (0.91 ± 0.23 mm). DIC revealed the highest maximum first principal strain around cortical button fixation (0.21%), followed by interference screw (0.16%) and keyhole (0.13%).

**Conclusion:**

Cortical button fixation demonstrated the highest load‐to‐failure and the lowest variability, indicating mechanical reliability. The interference screw and keyhole techniques showed comparable load‐to‐failure values and cyclic displacement but exhibited greater variability. DIC analysis revealed higher localized strain around the cortical button fixation, whereas the interference screw and keyhole techniques displayed more evenly distributed strain.

**Level of Evidence:**

Level V.

**Abbreviations**
DIC: digital image correlation
UTM: universal testing machine

## INTRODUCTION

Subpectoral biceps tenodesis is a common procedure designed to relieve pain and restore function by securing the long head of the biceps tendon [9]. Among the available fixation techniques, intramedullary cortical button fixation and interference screw fixation are commonly employed due to their excellent biomechanical properties [6, 19, 24, 33]. The keyhole technique, while less frequently used today, was primarily utilized when modern implants were not readily available [11]. Each of these methods offers distinct benefits; however, concerns remain regarding their mechanical reliability, potential for hardware failure and the reproducibility of outcomes.

Mechanical strength and durability are critical to successful tenodesis, as hardware failure can lead to fixation loss, increased pain or even the need for revision surgery [1]. Cortical buttons, interference screws and keyhole techniques vary in their load‐bearing capacity and in the way they transfer forces to the surrounding bone, directly impacting their mechanical stability and failure risk [6, 19]. A reliable evaluation of these techniques requires not only testing of their fixation strengths under various load conditions but also a reproducible analysis of how each technique withstands stress over time [33]. Clinical outcomes for subpectoral biceps tenodesis are typically good to excellent, with consistent pain relief and functional gains [22, 29]. However, complications have been reported, including implant failure, bioabsorbable screw reactions, persistent pain and humeral fractures, though the overall complication rate remains low at approximately 2% [23]. Several experimental studies have investigated the biomechanics of subpectoral biceps tenodesis; [6, 18, 24] however, none have directly compared cortical button, interference screw, and keyhole fixation techniques while incorporating digital image correlation (DIC).

The aim of this study was to conduct a detailed experimental mechanical assessment comparing cortical button fixation, interference screw and keyhole techniques in subpectoral biceps tenodesis, focusing on the frequencies of hardware failure and the mechanical resilience each technique provides. Additionally, DIC was employed to assess strain distribution around the bone drill site, offering deeper insight into fixation reliability and bone failure risks.

## MATERIALS AND METHODS

### Experiment design

A custom‐built apparatus was used to secure the porcine humerus within the lower grip of a universal testing machine (UTM). The distal portion of the tendon was attached, and axial loading was applied at a rate of 450 mm/min. A preload of 10 N was set, followed by cyclic loading ranging from 10 to 100 N for 500 cycles. The experiment utilized a Zwick Z020 UTM (ZwickRoell) to load the specimen with a low‐force cell with a range of up to 1 kN.

A DIC commercial system (software Alpha 2024.0.4, X‐Sight s.r.o.) was employed for measurement, integrating two Teledyne FLIR BFS‐U3‐88S6M‐C Complementary Metal Oxide Semiconductor digital cameras (8.9 Mpx, 4096 × 2160 resolution, 32 FPS, 3.45 µm pixel size), Sony IMX267 sensor (format 1 in., USB 3.1 Gen 1 interface, monochrome spectrum) alongside two entocentric lenses Computar V5028‐MPY [32]. The sampling frequency was set at 15 Hz. A subset size of 101 px (2.96 mm) was used for line measurements, while a subset size of 51 px (1.50 mm) was selected for DIC area analysis, with a step size of 0.2 mm. Both measurements utilized an affine subset shape function.

**Figure 1:**
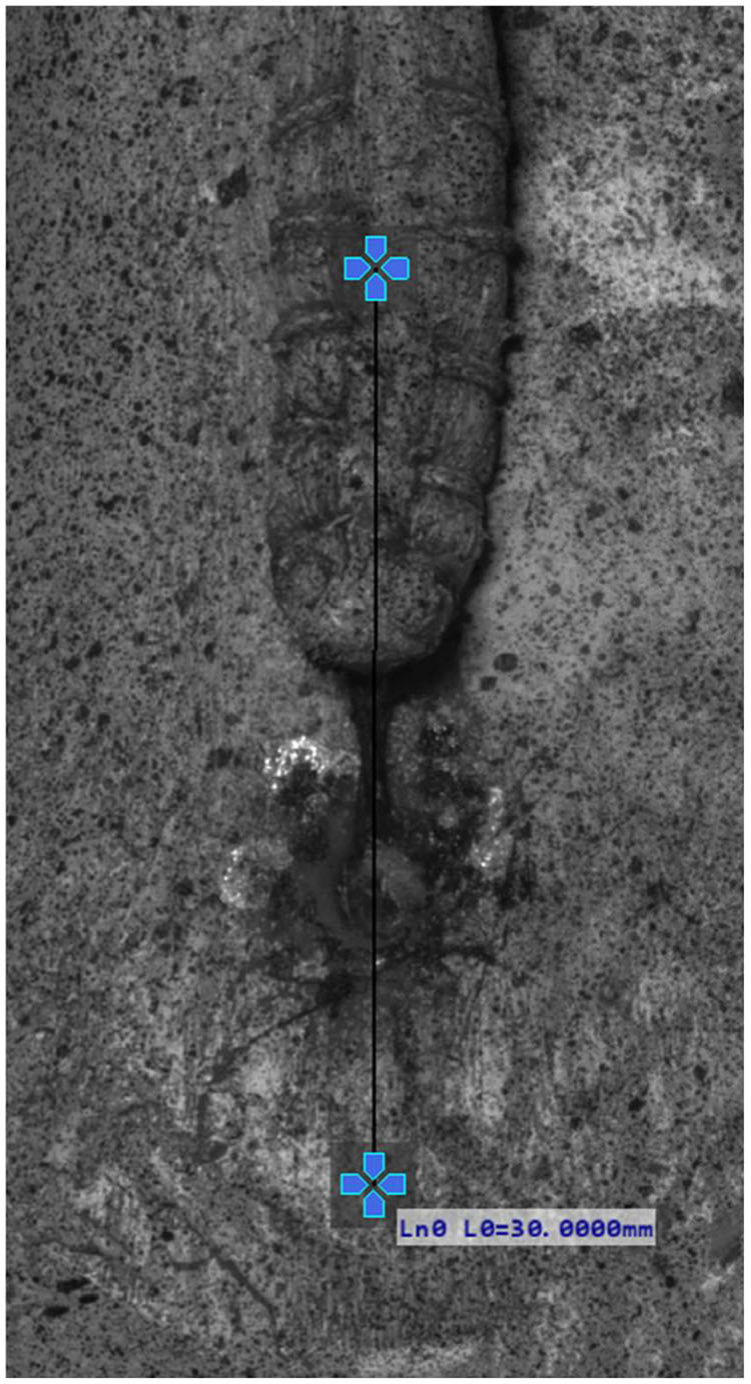
Line probe setup.

Displacement was calculated using a virtual strain gauge (line probe) based on the Lagrangian formulation, with an initial gauge length of 30 mm (Figure 1). B‐spline interpolation was applied, and the normalized sum of squared differences served as the matching criterion. Optical measurements were measured according to the ISO 9513 standard, accuracy class 0.5 [14]. The field of view was 120 mm × 52 mm, with a stereo angle of 15°, an aperture of f/10, a camera distance of 115 mm and a working distance of 430 mm. On all specimens, the speckle pattern was applied right before the measurement using acrylic black and white spray.

### Specimen preparation and surgical techniques

**Figure 2:**
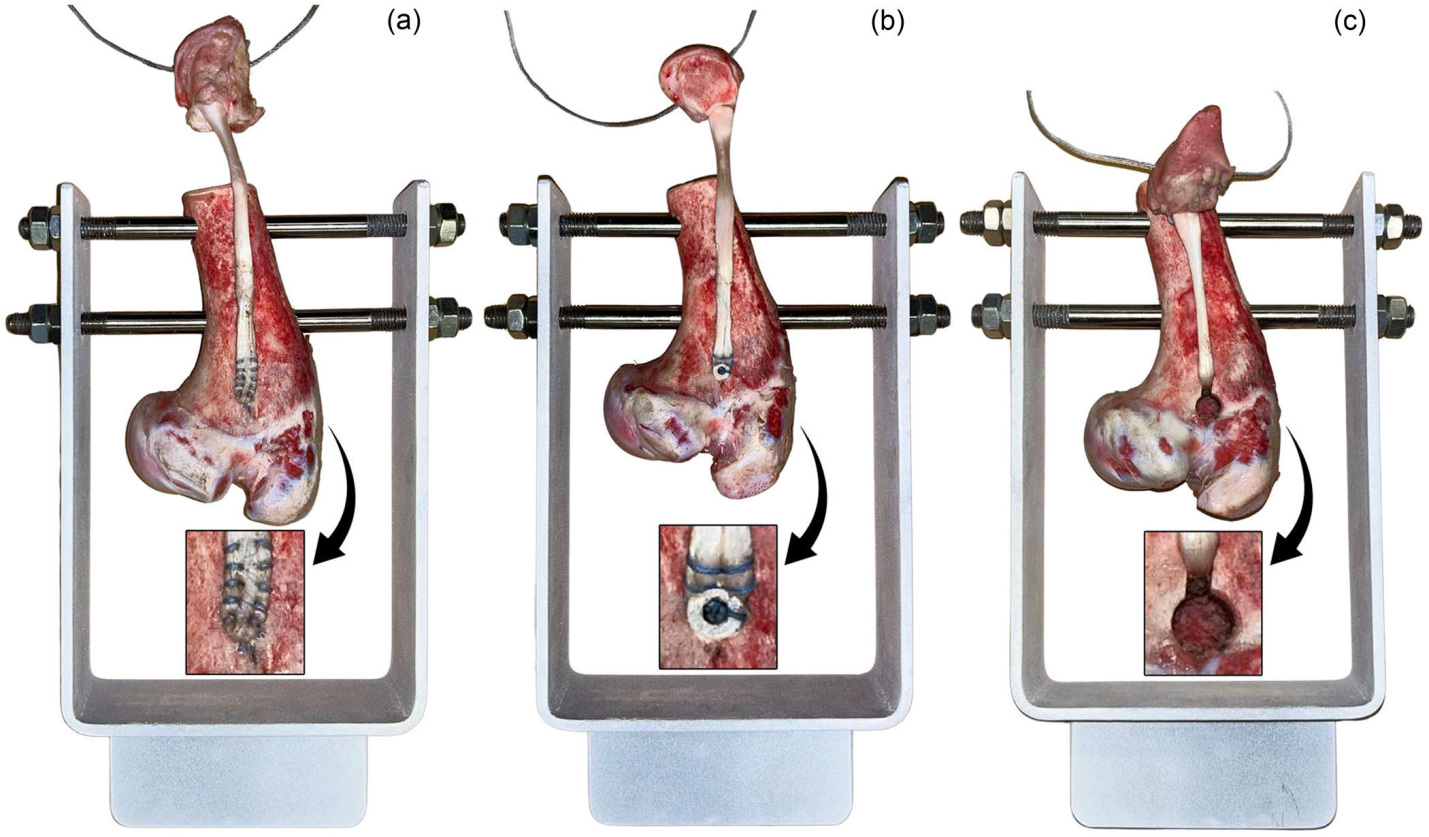
Specimen example for each fixation method in the fixation apparatus. (a) Cortical button, (b) interference screw and (c) keyhole fixation.

The organic materials used for this study were porcine humerus bones and tendons sourced from slaughter animals of uniform age and weight, obtained from certified slaughterhouses (Spektrum, spol. s.r.o.). The specimens were obtained from animals slaughtered for food production purposes, which were not genetically modified and were not euthanized specifically for this research. All animals were approximately 6 months old and weighed between 100 and 120 kg. To ensure consistency and comparability across groups, anatomical measurements were conducted on all specimens. The porcine long head biceps tendon was initially considered; however, we selected and modified the porcine flexor digitorum profundus tendon with distal phalanx as an alternative. The tendon fixation point was selected based on proportional anatomical landmarks in the porcine humerus, corresponding to the bicipital groove region described by Kusma et al., and positioned to ensure adequate medullary depth for secure fixation [18] (Figure 2).

A total of 30 fresh porcine cadaveric bone–tendon specimens were evaluated, with 10 assigned to each method of tendon fixation. The storage of these materials in a frozen state was avoided due to the potential alteration of the biomechanical properties of bone and tendon. Instead, biological materials were freshly collected for each testing series on the same day. Randomization was performed using a computer‐generated list of random numbers to ensure unbiased group allocation. All specimens were obtained from the same side (right). There were no statistically significant differences between the groups in terms of medullary and tendon diameters, as well as cortical thickness (*p* > 0.05). Measurements were obtained using a digital calliper (Digimatic Calliper, Mitutoyo, model 500‐184‐30). The mean cortical thickness of the bone in the bicipital groove (drill site) was 5.3 ± 0.5 mm. The anteroposterior diameter of the medullary space was 20.3 ± 1.3 mm, while the lateromedial diameter measured 26.1 ± 1.8 mm. The mean thickness of the tendons was 6.2 ± 0.3 mm, similar to the human long head biceps tendon [9]. The specimens measured 11.47 ± 0.60 cm from the origin of the bicipital groove.

Bones were dissected and carefully cleaned of soft tissue debris before being mounted in custom‐made holders. Tendons were also dissected from the limbs, ensuring the distal phalanx insertion was preserved. The samples were then maintained separately in a humidified environment with 0.9% saline solution at 4°C until testing. Testing for each group (10 specimens) was conducted at room temperature. This method ensured preservation of the biological material and reliable biomechanical testing conditions.

#### Cortical button fixation

The Proximal Tenodesis Button (Arthrex), a titanium cortical button, was employed [4]. The button, measuring 2.6 × 8.5 mm, features two holes designed for fibre insertion, providing a robust anchoring system. The porcine tendon of the deep flexor digitorum was prepared using five self‐locking Krackow sutures [17], placed 15 mm from the tendon end. No. 2 FiberWire (Arthrex) was utilized for the sutures.

The implant insertion site was chosen 20 mm distal to the proximal edge of the bicipital groove. A 3.2 mm original drill bit (Arthrex) was used to create a perpendicular hole into the spongiosa, just below the inner cortical bone. To ensure proper positioning of the button for unicortical fixation, a curved arthroscopic hook was introduced through the drilled hole to create a space in the spongiosa beneath the cortex. One end of the fibre was passed through both holes of the button in one direction, and the other end was introduced through the same holes in the opposite direction. This threading technique ensured the button would buckle securely under the inner cortex when the threads were tensioned. After the tendon was tied and the hole prepared, the cortical button was inserted into the drilled site using the original Arthrex Inserter. The inserter was disengaged, and alternate pulling of the fibre ends caused the button to buckle under the cortex securely, tensioning the tendon in the process. Finally, the suture was not passed through the tendon, and both fibre ends were secured with five knots to provide stable fixation [33].

#### Interference screw

The Tenodesis Screw (BioComposite, 7 × 10 mm, Arthrex) was employed as the interference screw in this experiment [5]. The implant insertion site was identical to that used for the cortical button. A 7 mm drill bit was utilized to create a hole matching the diameter of the interference screw, drilled perpendicular to the cortical bone to ensure proper alignment. For tendon preparation, the final 20 mm of the tendon was used, and the No. 2 FiberWire (Arthrex) was employed for reinforcement. Five whipstitch sutures were performed to secure the tendon firmly.

One end of the FiberWire was passed through the center of the interference screw, while the other end was left free, positioned between the screw and the bone wall. The FiberWire ends were fixed into the original Arthrex Inserter, ensuring that the tendon was securely attached to the screw's top. The screw was then carefully inserted and advanced, embedding the tendon into the hole. The head of the screw was positioned flush with the external cortex to achieve proper fixation. After securing the screw and tendon, the free ends of the FiberWire were tied using five knots to reinforce the attachment. This method provided a fixation, replicating clinical use of the Tenodesis screw in tendon repair procedures [16, 20].

#### Keyhole technique

The keyhole technique, frequently used in periods without orthopaedic implants, as described by Froimson and O [11], was the third technique in this study. The tendon was prepared by rolling its end into a compact sphere and securing it with multiple individual stitches to prevent unrolling during testing. The No. 2 FiberWire (Arthrex) was employed for this process, with firm single stitches used to tightly secure the rolled tendon. The resulting tendon sphere had a diameter of 10 mm, ensuring stability and consistency for implantation. The implantation site was identical to that used for the cortical button and interference screw techniques. A hole was drilled perpendicular to the cortical bone using a 10 mm diameter drill bit, and a keyhole notch measuring 7 mm in length and 3 mm in width was created along the long axis of the bone. This notch provided a secure groove for anchoring the tendon sphere to enhance fixation. A curved arthroscopic hook was employed to create additional space in the spongiosa beneath the cortical bone, facilitating firm anchorage. Once prepared, the tendon sphere was carefully inserted and distally positioned into the keyhole notch. Its placement was subsequently verified to ensure secure fixation. This method allowed for reproducible tendon anchorage, aligning with established keyhole technique principles.

### Evaluation

The line probe evaluated cyclic displacement relative to a 10 N preload throughout the testing period [18]. Load‐to‐failure was assessed after 500 cycles [6]. If the failure occurred before reaching the 500 cycles, the load‐to‐failure was set to 100 N [18, 33]. Load‐to‐failure was considered to have occurred when the testing machine stopped at a force drop of 50% from the applied maximum force. In the full‐field DIC, the evaluated parameter is the first principal strain, with percentage values.

### Statistical methods

Statistical analysis was conducted using R software (version 4.4.2) (R Foundation for Statistical Computing) within the RStudio integrated development environment. The mean amplitude was calculated as the average of the minimum and maximum values for each cycle. For each fixation type, the overall mean was determined by averaging the results across all specimens within the same fixation group. The displacement range, defined as the difference between the maximum and minimum displacement within a single cycle, was computed for each cycle. The mean displacement range was then calculated by averaging the displacement ranges across all tests for specimens within the same fixation group. A one‐way ANOVA was employed to compare the mean displacement and the mean load‐to‐failure across the three fixation methods. Specimens that failed to complete 500 cycles were excluded from the calculations of average displacement, average load‐to‐failure, and average displacement range. A *p* value of less than 0.05 was considered statistically significant.

## RESULTS

### Mean amplitude

**Figure 3:**
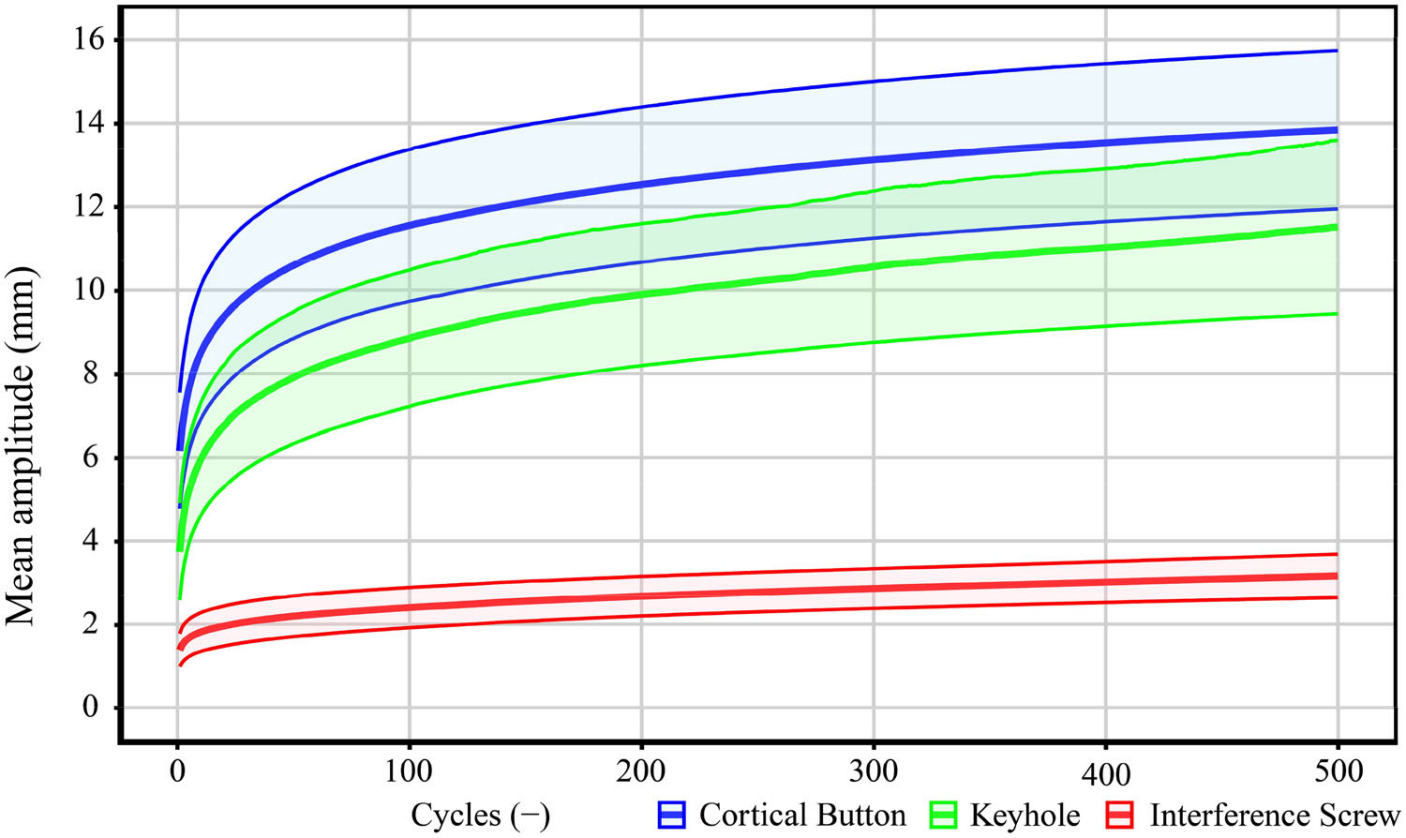
Average displacement with standard deviation for each type of fixation method.

Figure 3 shows the mean amplitude with standard deviation for each evaluated type of fixation. The initial mean amplitude during the first cycle was lowest for the interference screw fixation, measured at 1.38 ± 0.39 mm. After 100 cycles, the mean amplitude increased to 2.40 ± 0.48 mm (a 74% increase compared to the initial cycle), and after 500 cycles, it reached 3.16 ± 0.52 mm (a 32% increase compared to 100 cycles). For the keyhole fixation, the mean amplitude in the first cycle was 3.74 ± 1.16 mm. After 100 cycles, the mean amplitude rose to 8.84 ± 1.63 mm (a 136% increase compared to the initial cycle), and after 500 cycles, it further increased to 11.51 ± 2.08 mm (a 30% increase compared to 100 cycles). The highest initial mean amplitude was observed in the cortical button fixation, with a value of 6.16 ± 1.39 mm. After 100 cycles, the mean amplitude rose to 11.55 ± 1.82 mm (an 87% increase compared to the initial cycle), and after 500 cycles, it increased further to 13.84 ± 1.90 mm (a 20% increase compared to 100 cycles). Using a one‐way ANOVA to compare the mean amplitude across the three fixation methods, the analysis revealed a statistically significant difference after 100 cycles (*p* < 0.001) and after 500 cycles (*p* < 0.001).

### Load‐to‐failure

**Figure 4:**
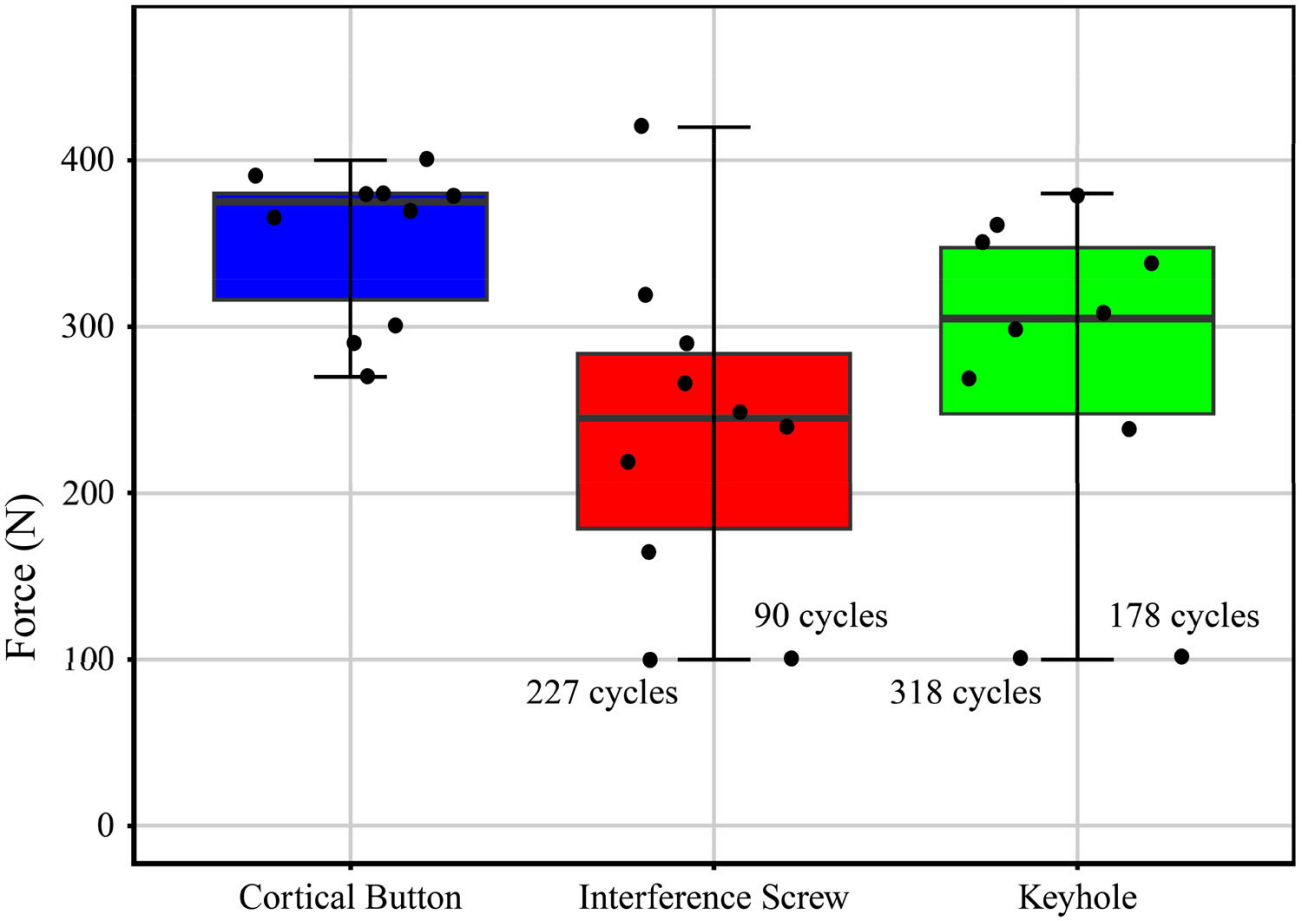
Load‐to‐failure for each type of fixation method.

Figure 4 shows the load‐to‐failure for each type of fixation. The average load‐to‐failure for the keyhole fixation was 319 ± 45 N, with two failures occurring before reaching 500 cycles. The lowest average load‐to‐failure presented a fixation with interference screw 271 ± 71 N with also two failures before completion of 500 cycles, and the highest load‐to‐failure of 353 ± 45 N had a cortical button fixation. Using a one‐way ANOVA to compare the mean load‐to‐failure across the three fixation methods, the analysis revealed a statistically significant difference (*p* = 0.018).

All implants in the cortical button cohort successfully completed the cyclic loading and load‐to‐failure tests. However, two failures occurred in the interference screw cohort during testing—one after 227 cycles and the other after 90 cycles. Both failures were caused by tendon slippage at the interface between the implant and the bone. Similarly, in the keyhole technique cohort, two failures occurred during testing—one after 318 cycles and the other after 178 cycles. In both cases, the mode of failure involved the unwrapping of the sphere.

### Displacement range

**Figure 5:**
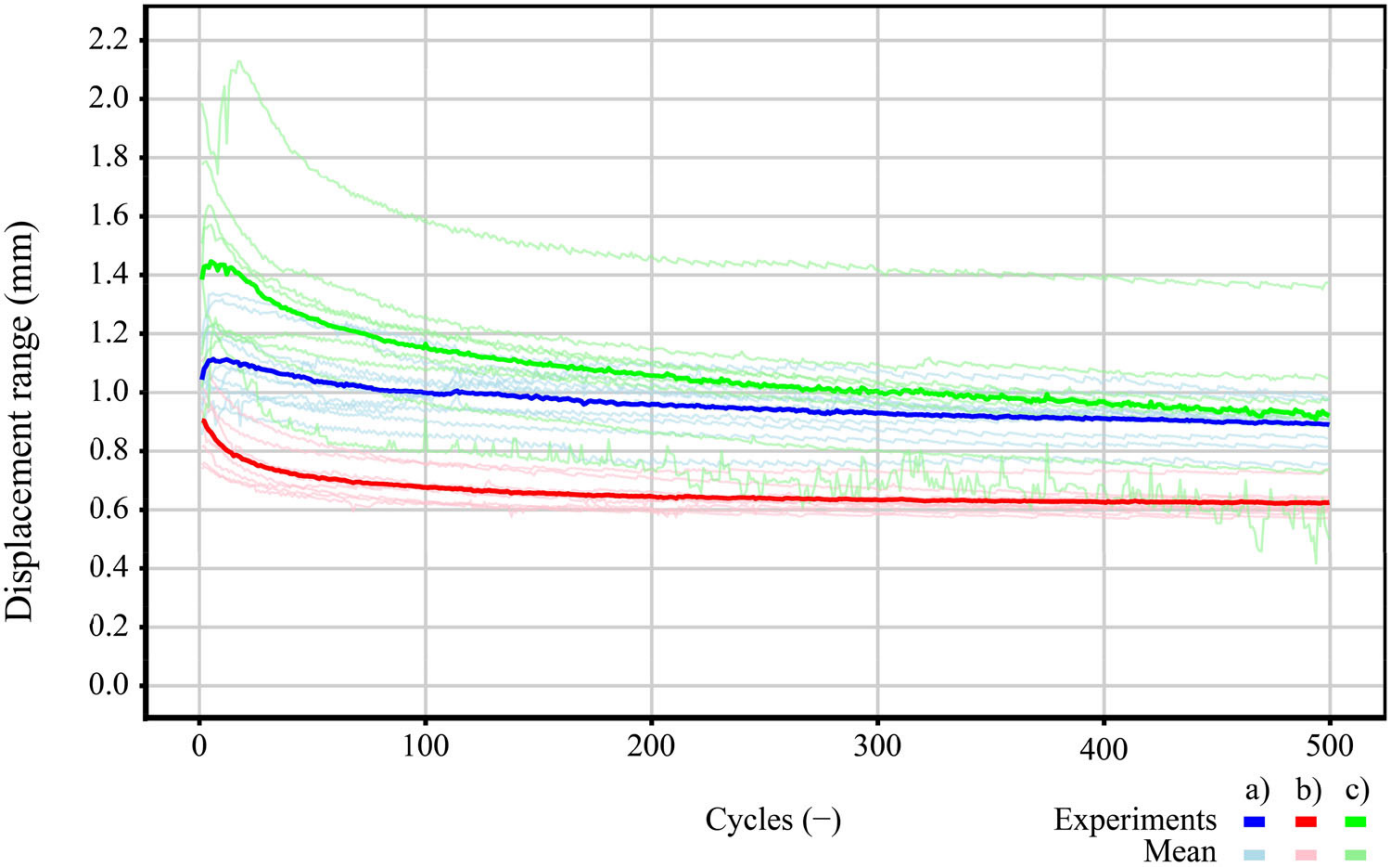
Displacement range with added mean for all fixation methods used: (a) cortical button, (b) interference screw and (c) keyhole fixation.

Figure 5 shows the displacement range for all fixation methods used. The average initial displacement range for cortical button was 1.04 ± 0.10 mm. After 100 cycles, the displacement range slightly decreased to 0.99 ± 0.09 mm, reflecting a 4.8% reduction compared to the initial displacement range. By 500 cycles, it further declined to 0.88 ± 0.07 mm, representing an 11.1% reduction compared to the displacement range at 100 cycles and a total 15.4% reduction from the initial displacement range (Figure 5). The average initial displacement range for interference screw was 0.90 ± 0.12 mm. After 100 cycles, it decreased to 0.67 ± 0.05 mm, representing a 25.6% reduction compared to the initial displacement range. By 500 cycles, the displacement range further declined to 0.62 ± 0.05 mm, showing a 7.5% reduction compared to the displacement range at 100 cycles and a total 31.1% reduction from the initial displacement range (Figure 5). The average initial displacement range for the keyhole technique was 1.38 ± 0.35 mm. After 100 cycles, the average displacement range decreased to 1.16 ± 0.20 mm, representing a 16% reduction compared to the initial displacement range. After 500 cycles, the average displacement range further decreased to 0.91 ± 0.23 mm, reflecting a 22% reduction compared to the displacement range at 100 cycles and a 34% reduction compared to the initial displacement range (Figure 5).

### Full‐field DIC area

**Figure 6:**
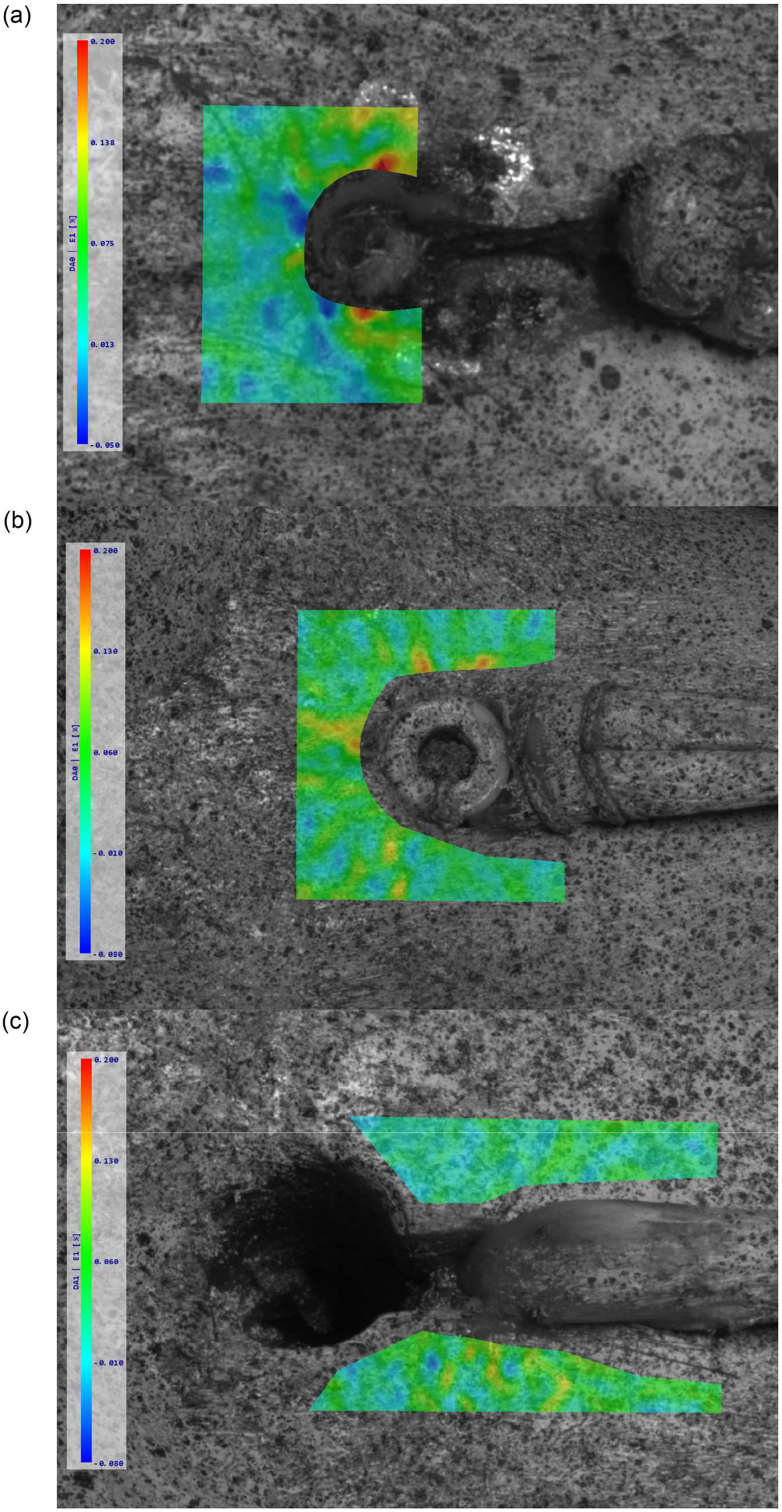
The first principal strain distribution on the surface of the cortical bone tissue for each type of fixation. (a) Cortical button, (b) interference screw and (c) keyhole fixation.

Figure 6 shows the full‐field strain analysis of the closest fixation area on the surface of the cortical bone. The cortical button fixation exhibited the highest maximum first principal strain at 0.21%, with strain concentration primarily localized around the cortical button's position in the prepared hole (Figure 6a). Compared to this, the interference screw fixation showed a lower maximum strain of 0.16%, representing a 23.8% reduction in strain relative to the cortical button fixation. Strain in the interference screw fixation was more evenly distributed around the screw area (Figure 6b). The keyhole fixation method exhibited the lowest maximum first principal strain at 0.13%, reflecting a 38.1% reduction compared to the cortical button fixation and an 18.8% reduction compared to the interference screw fixation. In the keyhole fixation, strain distribution was localized more towards the lower part of the fixation area (Figure 6c).

## DISCUSSION

Our study uniquely compares three specific fixation methods, acknowledging the variety of modern fixation techniques and implant options [18, 24]. We focused on mechanical properties—load to failure, displacement, and displacement range—after cyclic loading to enable comparison with recent literature. Cyclic loading simulated postoperative stresses on the bone–tendon–implant complex during rehabilitation [6]. Cortical button fixation offers superior mechanical reliability and reproducibility, but with higher localized strain. Interference screw and keyhole techniques demonstrate robust performance but are more sensitive to surgical technique. Notably, we are the first to use DIC to measure bone strain around the drilled hole and implant, adding a novel dimension not previously reported. These findings provide valuable guidance for clinical practice, aiding in the selection of fixation methods based on mechanical performance, reproducibility and strain distribution on the bone.

In our dataset, load‐to‐failure values after cyclic loading were recorded as follows: cortical button 353 ± 45 N, interference screw 271 ± 71 N and keyhole technique 319 ± 45 N. For the cortical button, load‐to‐failure values range from 99.4 to 348 N [3, 6, 7, 8, 19, 24, 33]. These variations likely reflect methodological differences, particularly in the type of tendon fixation stitches used (e.g., Krackow and whipstitch), which significantly influence construct strength [30]. The choice of the Krakow technique was based on its proven advantages, as demonstrated in previous studies, and aligns with the methodologies used in comparable experimental work [6, 24, 30]. For the interference screw, comparable values range from 191 to 276 N [6, 8, 21, 27, 33]. The use of a 7 × 10 mm screw in our study, reflects daily clinical practice, aligns with these findings, confirming that this screw size does not compromise performance. For the keyhole technique, our load‐to‐failure value (319 ± 45 N) closely matches Kusma's 320 N [18], while other studies reported 303 N [15] and 102 N [25]. The cortical button demonstrated the highest load‐to‐failure value and the smallest standard deviation, reflecting its ease of reproducibility when following standardized procedures. In contrast, the interference screw and keyhole techniques exhibited greater variability and were more sensitive to technical execution.

In the displacement analysis for cortical button fixation, reported mean displacements ranged from 8.4 to 29.2 mm [3, 8, 19, 24, 33]. Our results (mean displacement: 13.84 ± 1.90 mm) align with these reported ranges, with minor differences likely due to methodological variations. For example, we measured displacement optically directly on the tendon, while some studies used crossbar scales [6, 24]. Differences in cyclic loading protocols, such as maximum loads (Colantonio, Bucholz: 5–70 N; [6, 7] DeAngelis: 10–60 N [8]) or cycle counts (500 cycles vs. 200 cycles in Kusma [18]), also contribute to variability. Tendon fixation techniques further vary [6, 19, 24]. In our study, the fixation method mirrored clinical practice in our department. Supporting Bucholz's findings, we agree that fixation strength depends more on stitch type than the cortical button itself [6]. For interference screw fixation, our mean displacement was 3.16 ± 0.52 mm. Comparable values in literature range from 1.5 to 9 mm [3, 6, 18, 21]. These results confirm that our values are consistent with previously reported findings. For the keyhole technique, our mean displacement was 11.51 ± 2.08 mm. Published data are limited; Kusma reported 7.5 mm after fewer cycles (200 vs. 500 in our study) [18], while Jayamoorthy and Ozalay focused on load‐to‐failure values [15, 25].

The aim of our experiment was not to determine whether one method is superior but to provide a comprehensive analysis of subpectoral biceps tenodesis techniques, focusing on fixation types and their limitations. The interference screw offers excellent primary stability with minimal displacement; however, the load‐to‐failure is higher with other methods, notably the cortical button. While interference screw performance varies with size and diameter, our results align with existing data. Despite its reliability, the keyhole technique presents greater technical challenges, procedural complexity and reproducibility concerns, particularly in vivo. Both the keyhole and interference screw methods require larger humeral bone holes, which may increase the risk of spiral fractures, as reported in studies [10, 28, 31]. Conversely, cortical button fixation involves smaller bone holes, reducing fracture risk. Onlay unicortical fixation with a cortical button is a straightforward, stable and reproducible method. While it may exhibit slightly greater displacement, this can be minimized with proper stitch selection and technique, emphasizing the critical role of tendon fixation stability.

The use of DIC for full‐field strain analysis provided detailed and accurate insights into surface strain values and their distribution. In biomechanics, the DIC method is widely employed for analyzing strain and displacement, as evidenced by several studies [2, 26, 35]. Globally, DIC is most commonly recognized as a complementary technique to UTMs in material testing. It is used to measure deformation in standardized specimens, and due to its high accuracy, it facilitates the reliable determination of material properties. Furthermore, its capability for full‐field surface analysis provides valuable insights into strain distribution across sample surfaces, making it a powerful tool in both research and development applications. In our study, we analyzed the first principal strain around the bone drill site, resulting in values ranging up to 0.2% strain. These findings suggest mild overloading, which, according to Mechanostat, should promote bone modelling and remodelling in the fixation area [12, 13, 34, 36]. As such, the experimental strain values obtained can serve as reference data for future studies and offer a basis for comparison with computational models developed using the finite element method.

There are several limitations in this experimental study. First, the study employs isolated axial loading to simulate postoperative stresses, which does not fully account for the complexity of human shoulder kinematics, muscle group interactions or the varied load patterns experienced by the biceps tendon. Second, porcine tendons and bones were used as substitutes for human tissue, which, while providing a biomechanically similar environment, may differ in behaviour under physiological and long‐term conditions. Additionally, the analysis focuses on immediate and short‐term mechanical properties, such as load‐to‐failure and displacement after cyclic loading, without addressing long‐term factors like bone remodelling, healing or implant degradation. Furthermore, the controlled laboratory setting excludes critical real‐world variables, such as patient activity levels, biological responses and variations in surgical techniques, which could influence the outcomes. Conversely, the study employs standardized procedures for fixation techniques, including tendon preparation and implant placement, ensuring that the results are reliable and reproducible. Axial loading and cyclic testing closely simulate the mechanical demands placed on the tendon‐bone interface during rehabilitation. The use of freshly harvested biological materials, along with consistent storage and handling practices, ensures the preservation of biomechanical properties. Furthermore, the incorporation of DIC for strain analysis around the bone drill site is a novel approach, providing precise, high‐resolution insights into strain distribution that have not been previously reported.

## CONCLUSION

Cortical button fixation demonstrated the highest load‐to‐failure and the lowest variability, indicating mechanical reliability. The interference screw and keyhole techniques showed comparable load‐to‐failure values and cyclic displacement but exhibited greater variability. DIC analysis revealed higher localized strain around the cortical button fixation, whereas the interference screw and keyhole techniques displayed more evenly distributed strain.

## AUTHOR CONTRIBUTIONS

Lukáš Martinek was responsible for conceptualisation, specimen preparation, surgical technique, data interpretation, clinical validation, resources and manuscript writing. Petr Boháč performed research design, data and statistical analysis, data interpretation and manuscript preparation. Vasileios Apostolopoulos contributed to research design, clinical validation, data analysis, resources and manuscript preparation. Tomáš Návrat was responsible for data interpretation, research design, review and editing, and supervision. Róbert Langer and Luboš Nachtnebl contributed to the manuscript review and editing, and data interpretation. Tomáš Tomáš performed the formal analysis, project administration and supervision. All authors have read and approved the final submitted manuscript.

## CONFLICT OF INTEREST STATEMENT

The authors declare no conflicts of interest.

## ETHICS STATEMENT

The study was conducted according to the guidelines of the Declaration of Helsinki, approved by the St. Anne's University Hospital Ethics Committee (Brno, Czech Republic) (Protocol Code: EK‐FNUSA‐23/2024) and the Expert Committee for Ensuring the Welfare of Experimental Animals of the Faculty of Medicine of the Masaryk University.

## Data Availability

The data that support the findings of this study are available from the corresponding author upon reasonable request.
